# Sharing of Teaching Resources for English Majors Based on Ubiquitous Learning Resource Sharing Platform and Neural Network

**DOI:** 10.1155/2022/2683008

**Published:** 2022-06-08

**Authors:** Lijuan Zhang

**Affiliations:** Shanghai Jian Qiao University, Shanghai 201306, China

## Abstract

Teachers in traditional English education devote a great deal of time and effort to developing excellent English teaching courseware and other teaching materials. However, because the network's powerful sharing capability was not fully utilized, poor resource sharing hampered the school's overall teaching efficiency. The goal of this paper is to investigate how to use a ubiquitous learning resource sharing platform and a neural network to analyze the method of English major education resource sharing. Because the BP neural network algorithm has better classification and prediction functions, it was proposed because it can classify and predict information in the sharing of teaching resources for English majors. This allows for seamless sharing of teaching materials. According to the findings of this study, there were 562.13 million people learning English in China in 2015, accounting for 37% of the population. By 2018, 945.61 million people were learning English, accounting for 85 percent of the population. Almost every student is learning English, demonstrating the language's importance. As a result, the importance of English instruction is clear, and the sharing of teaching resources should be prioritized.

## 1. Introduction

Colleges and universities are educational institutions and departments with the most resources. The level of sharing of teaching resources for English majors in China is largely determined by the sharing of teaching resources for English majors. However, there are numerous issues with China's current resource construction and sharing that are impeding the growth of co-construction and sharing of teaching resources for English majors in China. As a result, the theoretical and practical implications of how to solve these problems and realize the co-construction and sharing of digital teaching resources among colleges and universities are significant. The resource sharing class provides rich resources, including video resources, but also these resources cannot be copied. Basic resources are provided for free, and teachers in different schools should be based on the actual situation of the school. They need to refer to relevant practices to construct courses suitable for their students and truly teach students in accordance with their aptitude.

The ubiquitous learning environment is a comprehensive learning environment produced from the physical, social, information, technological environment, and other aspects. In particular, various educational institutions, seminars, communities, and families are organically linked through networked devices. In addition, in a common learning environment, many scattered smart devices such as computers, mobile phones, and PDAs are used. Anyone can access the right source of information from any device at any time.

The innovation of this paper is that (1) it introduces related theoretical knowledge of English teaching resource sharing and neural networks, and proposes a neural network-based BP neural network (BPNN) algorithm. It examines the role of the BPNN algorithm in teaching resource sharing. (2) The effects of the BPNN algorithm and the other two algorithms on classification and prediction are compared and analyzed in this paper. The BPNN algorithm's classification and prediction effect are higher than the other two algorithms, as demonstrated by the experiment. This allows for data analysis in teaching resource sharing, resulting in increased efficiency.

## 2. Related Work

Rich and attractive teaching resources are the core link to make English professional education efficient. The sharing of teaching resources for English majors has begun to attract attention. Therefore, Yuan found that the intelligent network teaching system provides learners with rich teaching resources and an efficient learning environment. However, online teaching resources are widely distributed and difficult to concentrate. Resource sharing has become a key problem to be solved urgently in the network environment. Based on this, he studied link prediction methods in online education and builds a model suitable for online education. He used neural network sorting algorithm to realize online learning knowledge prediction [1]. Guichon proposed a method for creating multimodal corpora. This corpus can be shared to analyze simultaneous online teaching interactions. He described an analysis of the steps involved in creating a multimodal and shareable corpus [2]. Yan discovered that the university video management system has evolved into one of the most important video communication platforms. He created a microgrid-based teaching video resource management system to improve the storage and sharing of teaching video resource systems in colleges and universities. He realized video network storage and improved network sharing efficiency by constructing micro-technology network storage [3]. Tucker et al. found that one of the main difficulties in achieving effective online teaching was the difficulty of accessing the vast teaching resources of teachers. He also discovered the speech and language repository as a central repository for sharing, managing, and developing resources for science teaching [4]. The main purpose of Liu R's analysis of the fusion of network media and secondary English was to better play the function of secondary English. He discovered that using resource integration technology to manage distributed teaching resources helped colleges and universities improve their level of informatization [5]. Bai and Li designed a platform for statistical analysis of educational data and studied the storage of educational multidimensional data models in order to meet the rapid growth of educational data and improve operational efficiency and scientific decision-making. He then used educational big data to test query efficiency and storage space, demonstrating the utility of the Hadoop-based educational big data platform [6].

With the rapid advancement of education in recent years, teaching resources have expanded as well. Modern education is no longer limited to textbooks and individual teachers. Many developed countries encourage the sharing of educational resources, and many universities and primary and secondary schools in China are doing so as well. China's English professional education began late, but it has a brief history. Many aspects must be standardized and improved. As a result, traditional teaching resources are limited to teachers, and they are unable to meet the information-based teaching needs of modern society. Teacher resources can be shared to make teaching more efficient and the classroom more colorful.

## 3. Sharing of Teaching Resources for English Majors Based on Neural Network

### 3.1. Ubiquitous Learning Resource Sharing Platform Based on Web2.0 Environment

The importance of English is growing due to the informatization of social life and the globalization of the economy. English has become the most widely used language in all aspects of human life as one of the most important information carriers. English is now considered a necessary skill. If a student can learn a foreign language, he can open the door to a new world of learning and ultimately achieve the goal of multiple learning and value. With the progress of globalization, English is very important especially for those who want to communicate with foreigners. On the other hand, English is a necessary factor for understanding the outside world. In today's world, most of the communication of cultural confidence is achieved through the linguistic form of English, especially through high information [7]. The number and percentage of English learners in recent years are shown in Table 1.

**Figure 1:**
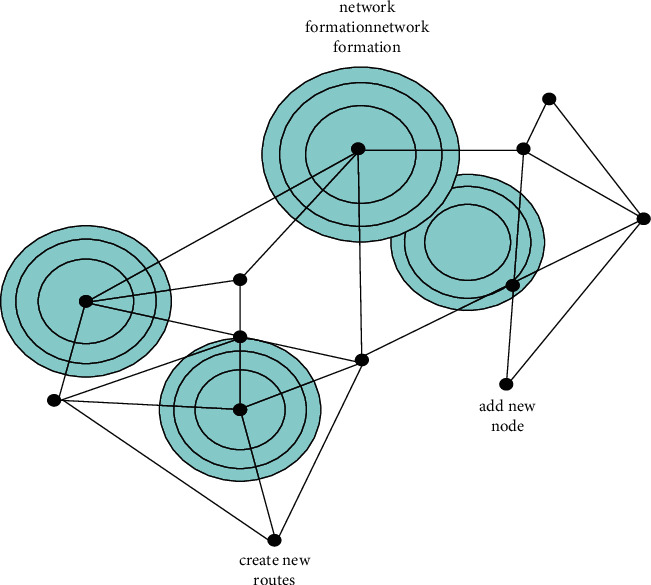
Ubiquitous learning network construction.

The number of people learning English is increasing, as shown in Table 1. The term “ubiquitous learning network” refers to a technology that allows users to freely access and exchange information over broadband and wireless networks at any time, from any location, using any tool. The Internet, digital TV networks, satellite networks, wireless networks, and 3G mobile communication networks are all part of it. The communication network is the backbone of the entire learning environment and is in charge of basic data transmission [8]. Intelligent terminals are linked together to allow for the transmission and sharing of information and resources, and they help to create a ubiquitous learning environment.  Figure 1 depicts the creation of the ubiquitous learning network.

As shown in Figure 1, ubiquitous learning is also referred to as seamless learning, pervasive learning, and ubiquitous learning, among other terms. It refers to all forms of communication and learning at all times. It is a way for anyone, anywhere, at any time, to get any information they require. In human learning, the important part of ubiquitous computing technology is to provide learners with a ubiquitous learning environment. The most obvious feature of a universal learning environment is the universality and conditionality of learning [9].

For example, a teacher may use a computer in the classroom to look for additional teaching materials, assign homework to students using a mobile phone, or better organize his teaching schedule. A learner in the park can use his phone to get information from his friends and classmates, such as learning tasks [10].

**Figure 2:**
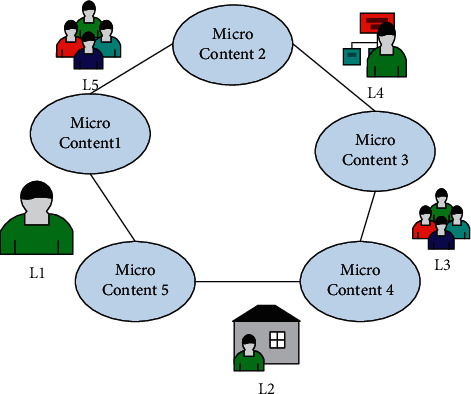
Web2.0 teaching resource sharing platform.

In recent years, China's scientific and information technology has developed faster and faster. Among them, Web2.0 and other related information technologies are used more and more widely, and the information resources are also more and more abundant. This also makes the information-based teaching constantly improved in recent years. From the point of view of system design and technology, Web2.0 has the characteristics of adaptability for user interaction, main participatory architecture, open architecture for other systems, multiple nonlinear mechanisms, and sociality [11]. The Web2.0 teaching resource sharing platform is shown in Figure 2.

Traditional network resource construction has some flaws, as shown in Figure 2. Meeting user needs, such as user participation, creating or adding content, forming a community, and so on, is insufficient. And some Web2.0 application commonalities can just about satisfy it. It facilitates communication between those who have resources and those who require them, and it makes network resources social and open [12]. The free sharing and integration of information resources is the source of Web2.0. To achieve this goal on the web, a mechanism and technology must be in place to support resource exchange and matching, and these mechanisms and technologies are sufficient to meet the needs of Web2.0 sites.

#### 3.1.1. Open API

**Figure 3:**
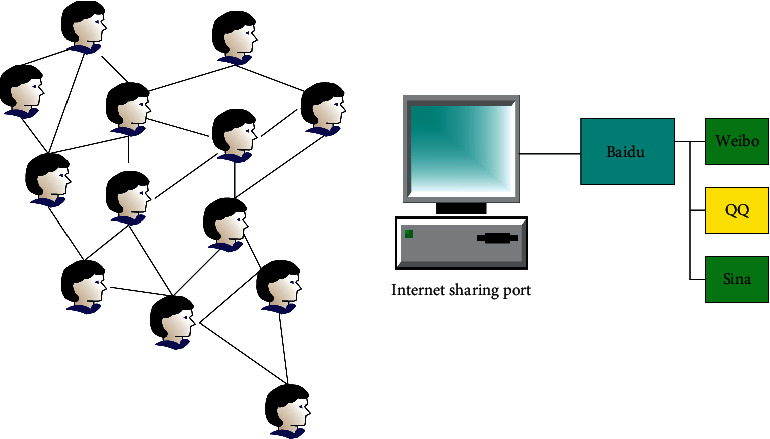
Internet sharing API.

API generally refers to an application programming interface. The main purpose of the API is to provide applications and developers with the ability to access a set of routines without having to access the source code or understand the details of the inner workings. Software that provides the functionality defined by an API is called an implementation of that API. The application of Web2.0 is becoming more and more popular, and this open architecture gradually emerges in the application process. From API to open API in other various websites and social software programmable web, the system pays more and more attention to the interaction and integration with other systems. Whether it is open and can form good writing with other systems becomes the key to whether web information resources can attract and be reused by users [13]. The Internet sharing API is shown in Figure 3.

As shown in Figure 3, the opening of all APIs makes the sharing, acquisition, and service of a large number of network information resources simple and easy, reducing the threshold of user experience. Simultaneously, these open platforms also bring greater value to users, developers, and small and medium-sized websites [14].

#### 3.1.2. RSS

RSS is a data exchange method and distribution protocol for individuals to share content among other websites and is an XML form for distributing and aggregating web content. RSS builds a technical platform for the rapid dissemination of information, making everyone a potential information provider. RSS is widely used in major websites and blogs. To realize the sharing and recompilation of information resources, its significance lies in the realization of machine readability of information content, synchronization, and integration of content between systems [15].

### 3.2. Problems Faced by the Sharing of Teaching Resources for English Majors

**Figure 4:**
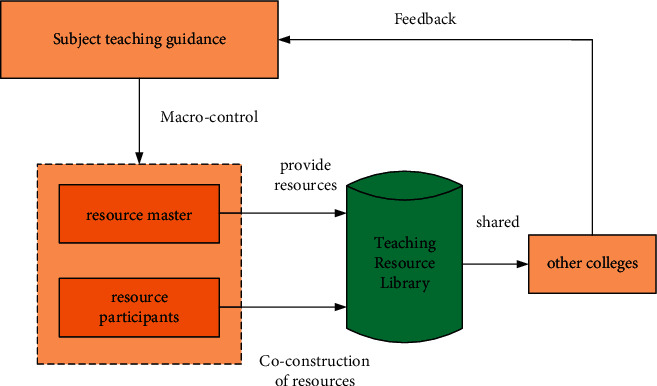
Shared structure diagram of subject teaching resource library.

The main resource builder should make detailed plans for the construction of course resources to ensure shared use of teaching resources for English majors. Participating in resource development should result in useful teaching materials [16]. A special English teaching resource library can be established to meet the needs of different teachers and students when it comes to this subject's teaching resources. Accessing the teaching resource library, as shown in Figure 4, allows college teachers and students to find and use the teaching resources they require.

As shown in Figure 4, prosperous English teaching resources have brought benefits to people. Not only colleges and universities themselves have made huge profits, but more importantly, college users can enjoy more and richer resources. It also provides an effective development method for the development of lifelong education and distance education in China [17]. But after years of resource construction, the consequences of blindness and disorder have become more and more obvious. It is mainly reflected in the following aspects:

1. Scattered resources, lack of normative and systematic
2. Many universities are now developing their own resources, and university students have access to some digital resources as well. However, the majority of these resources are dispersed, and many of those created by universities lack a unified classification standard and plan. The amount of resource data grows as the number of resources built increases, and the interface problem becomes more apparent. The sharing of digital educational resources has been greatly hampered, and the resource utilization rate has been significantly reduced, due to the lack of various resource technology platforms and tools to provide users with services.
3. The phenomenon of “information island” is serious, and repeated construction leads to a lot of waste
4. Because the interconnected functions and advantages are not fully utilized, and the human, material, and financial resources invested in the process of resource construction, universities are rarely willing to provide free resources to others. This will greatly limit resource sharing. Despite significant progress in the development of educational resources, there are still numerous issues. Co-construction resources are scarce, sharing coverage is limited, construction duplication is common, resource quality is poor, and resource inhomogeneity caused by platforms and data forms is on the rise. People should use BP neural networks [18] to better understand educational resource information classification [19, 20] and prediction [21], as well as to share educational resources more effectively [22].

### 3.3. The Classification Function of BP Neural Network

Large-scale parallel processing, decentralized storage and processing, self-organization, self-adaptation and self-learning ability, nonlinear approximation characteristics, and so on are all advantages of neural networks as one of the classic methods for dealing with classification and prediction problems. It is appropriate for dealing with inaccurate data, but there are a few things to consider. Retraining the network takes a long time when the trained learning system needs to learn new knowledge from new samples. On the premise of preserving the majority of previously learned knowledge, incremental learning can partially adjust network parameters to adapt to new knowledge.

**Figure 5:**
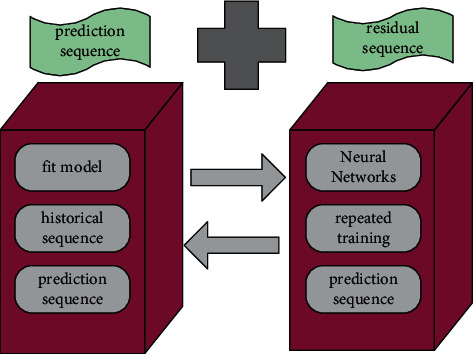
Fitting ability of BP neural network.

The BP neural network is a multi-layer feedforward network with error backpropagation training (referred to as error backpropagation). It uses the BP algorithm, which is based on the gradient descent method. The complex network structure and repeated learning process of BP neural network make it not affected by random or unknown factors, and it has outstanding fitting ability to complex chaotic systems. The fitting ability of the BPNN is shown in Figure 5.

**Figure 6:**
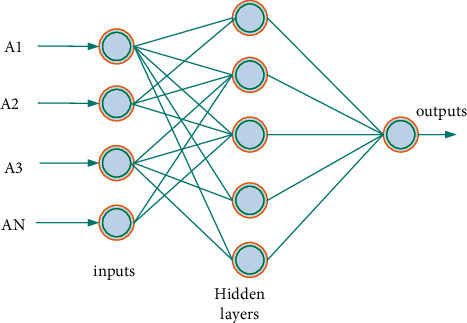
BP neural network structure diagram.

Figure 5 shows that the neural network has strong self-adaptive learning and parallel processing capabilities, but it is difficult to properly represent the input-output relationship obtained. One of the most widely used and successful artificial neural networks is the BP neural network model. It can be used in a variety of fields, including image and sound, due to its powerful nonlinear mapping capability. When the BPNN is applied to time series data, a large amount of data preprocessing is not required, allowing the previous time to be effectively reduced. As shown in Figure 6, the sequence classification algorithm is designed to improve classification performance while extracting features.

The number of nodes in the output layer, as shown in Figure 6, is set according to the needs of the actual problem. The number of nodes in the hidden layer determines the neural network's complexity. In general, the more nodes in the network's hidden layer, the more difficult the problem is to solve. A neural network is known to have *n* input layer nodes, *m* hidden layer nodes, and one output layer node. It can update the weights of each connection chain using formula (1) to describe the neural network's training process:

(1) $$\begin{array}{c} {\text{net}}_{j}=\sum_{i=0}^{n}v_{ij}a_{i},\;j=1,2,\ldots ,m. \end{array}$$

net_*j*_ represents the excitation value of the first node, *a*_*i*_ represents the output value of any node in the hidden layer, and *v*_*ij*_ represents the excitation function of the hidden layer node. It generally uses the sigmoid function, such as formula

(2) $$\begin{array}{c} f_{H}\left(a\right)=\frac{1}{1+\exp\left(-a\right)}. \end{array}$$

The output value of the output layer node is shown in formula

(3) $$\begin{array}{c} O=f_{0}\left(\sum_{j=0}^{m}w_{jk}b_{i}\right). \end{array}$$

*f*_0_ is the excitation function.

Compared with a single classifier or predictor, ensemble learning can achieve better performance and generalization ability. The main methods of ensemble learning include bagging, boosting, AdaBoost (adaptive boosting), and other algorithms. Bagging algorithm constructs different training sets by sampling samples with replacement, thereby generating different learners. It finally gets the final result by voting.

The principle of AdaBoost algorithm is to adjust the sample weight and weak classifier weight. It selects the weak classifiers with the smallest weight coefficients from the trained weak classifiers and combines them into a final strong classifier. The AdaBoost algorithm assigns different weights to different sub-models under the same training set. The algorithm first assigns the same initial weight to each input sample. In each round of iteration, it adds new base classifiers until a preset small enough accuracy is reached or all base classifiers are used for training to determine the final classifier.

AdaBoost. HM algorithm is an improved algorithm of AdaBoost algorithm. AdaBoost algorithm calculates the error of the classifier on the initial weight distribution by calculating the classification error rate of the base classifier. The AdaBoost. HM algorithm calculates the error of the classifier on the initial weight distribution by calculating the classification accuracy of the base classifier. Its calculation formula is

(4) $$\begin{array}{c} u\left(a_{z}\right)=h^{y_{z}}\left(a_{z}\right)-\max_{y}h^{y}\left(a_{z}\right). \end{array}$$

Among them, *z*=1,…, *Z* is the total number of input data, and *h*^*y*^ represents the class label data consisting of multiple 0 s and 1 s. The formula for calculating the weight of the base classifier in the final classifier is also different. The formula for the AdaBoost algorithm to calculate this weight is formula

(5) $$\begin{array}{c} \gamma =\frac{1}{2}\text{in}\left(\frac{1-r}{r}\right). \end{array}$$

The formula for the AdaBoost.HM algorithm to calculate this weight is

(6) $$\begin{array}{c} \gamma =\frac{1}{2}\text{In}\left(\frac{1+r}{1-r}\right). \end{array}$$

*r* is the error of the base classifier.

In this paper, improvements are made to the AdaBoost.HM algorithm. The classifier coefficients calculated by the formula are often not optimal. Therefore, people add a coefficient to the formula for calculating the weight coefficient of the base classifier in the final classifier and use the particle swarm optimization (PSO) algorithm to optimize the coefficient. People use the result of the base classifier as a kind of weight. It uses the direct output of the base classifier to compute the final classifier instead of converting it to class label data. It selects a classifier with the lowest current error rate as the first base classifier. It calculates the accuracy of this classifier as formula

(7) $$\begin{array}{c} u_{l}\left(a_{z}\right)=\left\{\begin{array}{cc} 1, & b_{z}=f_{l}^{z},\\ -1, & b_{z}\neq f_{l}^{z}. \end{array}\right. \end{array}$$

*f*_*l*_^*z*^ represents the actual output class of the *l*th base classifier relative to input *a*_*z*_. The error of this base classifier on the *d*_*l*_-distribution is formula

(8) $$\begin{array}{c} r_{l}=\sum_{z=1}^{Z}d_{l}\left(z\right)u_{l}\left(a_{z}\right). \end{array}$$

The weight of the base classifier in the final classifier is calculated as formula

(9) $$\begin{array}{c} r_{l}=p\text{In}\left(\frac{1+r_{l}}{1-r_{l}}\right),\;p\in \left[0,1\right]. \end{array}$$

It updates the weight distribution of training samples as formula

(10) $$\begin{array}{c} d_{l+1}\left(z\right)=\frac{d_{l}\left(z\right)\exp\left(-\gamma_{l}u_{l}\left(a_{z}\right)\right)}{\sum_{z=1}^{z}d_{l}\left(z\right)\exp\left(-\gamma_{l}u_{l}\left(a_{z}\right)\right)}. \end{array}$$

It combines each base classifier according to the weight of the base classifier to obtain the final classifier as formula

(11) $$\begin{array}{c} H\left(A\right)=\arg\;\max_{o}\sum_{l=1}^{M}\gamma_{l}H_{l}^{o}\left(A\right). \end{array}$$

*H*_*l*_^*o*^(*A*) represents the direct output of the lth base classifier.

### 3.4. Naive Bayes Algorithm

The Bayesian method is based on the Bayesian principle and classifies the sample dataset using probability and statistics knowledge. The Bayesian classification algorithm has a very low false-positive rate due to its solid mathematical foundation. The principle of the Naive Bayes algorithm is simple. It assumes that the various characteristic attributes of the sample have independent effects on the classification of the sample. It can more accurately estimate the necessary parameters when the amount of data is small. In addition, the classification model is characterized by simple installation, fast classification speed, and high accuracy.

The training process of the Naive Bayes classifier is to estimate the class prior probability *P*(*c*) based on the training set *D*.

(12) $$\begin{array}{c} P\left(c\right)=\frac{\left|D_{C}\right|}{\left|D\right|}. \end{array}$$

It estimates the conditional probability that the *i*th feature in the training sample appears in class *c* as formula

(13) $$\begin{array}{c} P\left(a_{i}|c\right)=\frac{D_{c,a_{i}}}{D_{c}}. \end{array}$$

Although the Naive Bayes classifier is simple and efficient, when the labeled data are small, the number of samples much larger than the training will cause the problem of over-adaptation, which will lead to insufficient classification effect.

### 3.5. Prediction Model of Teaching Resource Sharing Based on BP Neural Network

Using neural network as the base classifier or predictor of ensemble learning can improve the generalization ability of the network and the effect of classification or prediction.

Prediction usually works through classification or valuation. That is, predictions are usually modeled by classification or estimation, and the models are applied to predictions of unknown variables. The target of prediction is to predict the unknown variables in the future, which often takes time to verify. The process of modeling and training using neural network is as follows:

Since the impact factor units are inconsistent, people first normalize the data to the [−1, 1] interval. Its formula is

(14) $$\begin{array}{c} a_{k}=\frac{a_{k}-a_{\min}}{a_{\max}-a_{\min}}. \end{array}$$

The output of the BP rule in the BP wavelet neural network is the wavelet function. The network not only has the self-learning and self-adaptive ability of BP neural network, but also has the characteristics of wavelet function. In this section, the wavelet function used is expressed as formula

(15) $$\begin{array}{c} \varphi_{il}\left(a_{i}\right)=-\zeta_{il}\exp\left(\frac{-\zeta_{il}^{2}}{2}\right). \end{array}$$

*ζ*_*il*_ is the translation factor and scaling factor of the wavelet function.

The first layer is the input layer, and the *i*th output can be expressed as formula

(16) $$\begin{array}{c} f_{1}\left(a_{i}\right)=a_{i},\;i=1,\ldots ,n. \end{array}$$

The second layer is the pooling layer. Each node of this layer is used to calculate the membership function of the BP subset corresponding to input variable *a*_*i*_. In this section, the Gaussian BP function is used to input the neural network, and the output of the neuron node of this layer can be expressed as formula

(17) $$\begin{array}{c} \mu_{il}=\exp\left(-\frac{{\left(a_{i}-m_{il}\right)}^{2}}{2\sigma_{il}^{2}}\right),\;\left(i=1,\ldots ,n\right). \end{array}$$

The third layer is the regular aggregation layer. It uses the multiplication operator to calculate the membership of each rule. The output expression of this layer is formula

(18) $$\begin{array}{c} \mu_{l}=\prod_{i=1}^{n}\mu_{il}. \end{array}$$

The fourth layer is the normalization layer, whose main function is to normalize the membership degree of each BP rule to formula

(19) $$\begin{array}{c} v_{l}=\frac{\mu_{l}}{\sum_{l=1}^{L}\mu_{l}}. \end{array}$$

Here are 0 < *v*_*l*_ ≤ 1 and ∑_*l*=1_^*L*^*μ*_*l*_=1.

This paper uses a learning rate based on the idea of variable universe for parameter learning. This paper uses the classification accuracy of the network with the training sample set to construct the scaling factor. It can adaptively adjust the size of the learning rate according to the training effect of the network. Its expression is as formula

(20) $$\begin{array}{c} \eta \left(k\right)={\left(\frac{100-\text{accuracy}\_\text{train}\left(k-1\right)}{\text{acc}\_\text{const}}\right)}^{T}\eta \left(k-1\right). \end{array}$$

Among them, *k* is the number of iterations, and 100 − accuracy_train(*k* − 1) is the percentage value of the classification accuracy of the training set samples predicted by the network after the last iteration.

When the values of acc_const and *T* are determined, it is not difficult to see from the expression of the scaling factor that at the beginning of the iteration or when the number of iterations is small, the value of the model classification accuracy 100 − accuracy_train(*k* − 1) is very small, and the value of *T* is greater than 1. The learning rate for the *k*-th iteration is increased relative to the previous iteration. This can improve the global search ability of the model and speed up the network convergence speed.

Before dealing with the time series forecasting problem, it first divides the time series data into training set and test set. It converts the predicted input and output data into a matrix representation. It is assumed that the first *s* sequence values are used to predict the sequence value at the next moment, and the formula is

(21) $$\begin{array}{c} \left[\begin{array}{ccc} a_{1} & \ldots & a_{s}\\ a_{t} & \ldots & a_{t+s-1}\\ a_{T} & \ldots & a_{T+s-1} \end{array}\right]⟶\left[\begin{array}{c} a_{s+1}\\ a_{t+s}\\ a_{t+s} \end{array}\right]. \end{array}$$

Matrix *A* represents the input to the model, *a*_*s*+1_ represents the output of the model, and *T* is the number of rows in matrix *A*. The arithmetic mean of the results of the two base predictors is directly taken as the integrated prediction result; that is, each base predictor is given a weight of size *N*_*f*_ and then weighted and summed. The integration result is as formula

(22) $$\begin{array}{c} b_{\text{ensemble}}=\frac{\sum_{l=1}^{N_{f}}b_{i}}{N_{f}}. \end{array}$$

*b*_ensemble_ is the result after ensemble, *b*_*i*_ is the result based on the lth predictor, and *N*_*f*_ is the number of base models participating in the ensemble.

## 4. Experiment and Analysis of Classification and Prediction of BP Neural Network

### 4.1. Classification Function Experiment of BP Neural Network

In the experiments, 9 sets of time series data were selected from various data sources in the UCR time series classification software house. The specific information of the experimental data selected from the UCR time series classification software house is shown in Table 2.

As shown in Table 2, the BPNN constructed in this paper has a three-layer structure. The amount of nodes in the input layer is the data length of the time series data, and the number of nodes in the output layer is the number of data categories.

**Figure 7:**
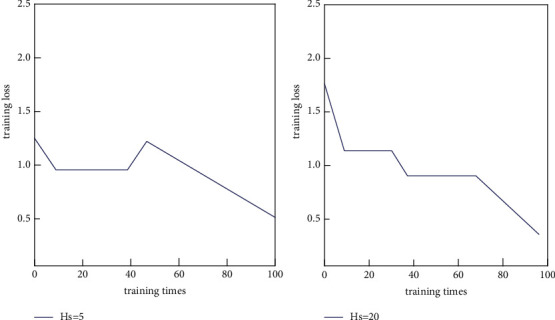
Training loss of BP neural network on Beef dataset.

The setting of neural network training time usually needs to be manually adjusted according to the experimental results. If there is insufficient labeled data, the training loss of the neural network may converge faster due to fewer training samples. The training loss of the neural network in the datasets Beef and Gun-Point is shown in Figure 7.

**Figure 8:**
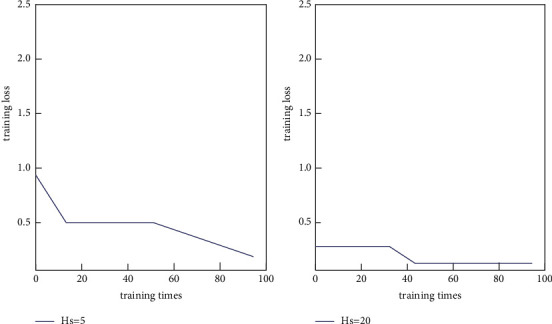
Training loss of BP neural network on Gun-Point dataset.

As shown in Figures 7 and 8, the neural network's training time setting must usually be manually adjusted based on the experimental results. A neural network is a distributed parallel information processing algorithmic mathematical model that mimics the behavioral characteristics of animal neural networks. Due to fewer training samples, the neural network's training loss may converge faster if there are insufficient labeled data.

For the above 9 datasets, the proposed BP classification algorithm is compared to the conventional SVM classification algorithm and the simple Bayes algorithm in this paper, as well as the classification effect of the classifier using only a limited number of labels. The accuracy of classification is used to assess the strengths and weaknesses of various algorithms. The best classification results of various parameter settings are BPNN classification algorithm, support vector machine classification algorithm, and Naive Bayes algorithm.  Table 3 illustrates this.

**Figure 9:**
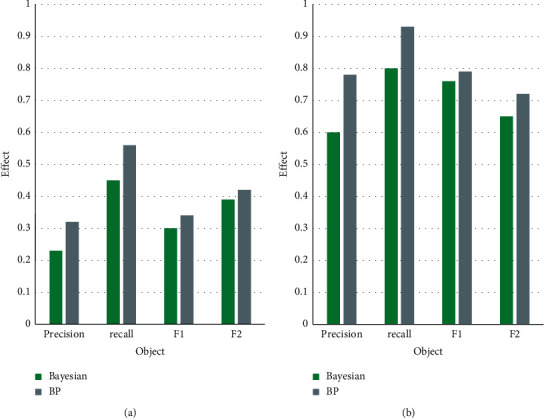
Comparison of the classification effects of the two algorithms on different datasets. (a) Comparison of classification effect of Beef dataset. (b) Comparison of classification effect of Gun-Point dataset.

Table 3 shows the following: the highest accuracy is 0.964 for the BP neural network classification algorithm, while the highest accuracy is 0.809 and 0.926 for the support vector machine classification algorithm and the Naive Bayes algorithm, respectively. The BPNN classification algorithm outperforms the support vector machine and Naive Bayes classification algorithms. The best classification effect is achieved by the BP algorithm proposed in this paper. The BP algorithm and the Naive Bayes algorithm proposed in this chapter are used as benchmark classifiers in this experiment. It expands the label set with the AdaBoost algorithm and compares the classification effects by building a temporal classification model.  Figure 9 depicts the experimental results.

As shown in Figure 9, in the Beef dataset and Gun-Point dataset, if the BP algorithm proposed in this paper is used as the benchmark classifier, the simple Bayes algorithm has matching rate, recurrence rate, and metric values. If the BP algorithm proposed in this paper is used as a benchmark classifier, unlabeled data can be labeled more accurately. The AdaBoost algorithm can take into account unlabeled data and make better use of unlabeled data information for improvement.

### 4.2. Prediction Experiment and Analysis of BP Neural Network

This part compares the accuracy of the prediction model through experiments and evaluates the performance of English major teaching resource sharing prediction based on BP neural network. In the following comparative experiments, it selects the BPNN prediction model and the Naive Bayesian model as the experimental comparison object and selects the error index of the prediction sequence as the model evaluation standard.

In order to better explain the experimental results, on the one hand, it shows the error rate of the prediction results, and on the other hand, it visually shows the fitting effect and prediction results of each prediction model to the experimental data. In general, it evaluates the performance of forecasting models according to the following three generally applicable error measures: mean absolute error (MAE), mean percentage error (MAPE), and root mean square error (RMSE).

(23) $$\begin{array}{c} \text{MAE}=\frac{\sum_{i=1}^{n}\left|b_{t}-b_{l}\right|}{n},\\ \text{MAPE}=\frac{\sum_{t=1}^{n}\left|b_{t}-b_{l}\right|}{n\times \left|b_{t}\right|}\times 100\%,\\ \text{RMSE}=\sqrt{\frac{\sum_{t=1}^{n}{\left(b_{t}-b_{l}\right)}^{2}}{n}.} \end{array}$$

Among them, *b*_*t*_ is the actual value of the time series at time *t*, and *b*_*l*_ is the predicted value of the time series at time *t*.

This experiment is to compare the Naive Bayesian model and the BP neural network prediction model. The datasets are the data of teaching resources of English majors in College A and the data of teaching resources of English majors in College B.

**Figure 10:**
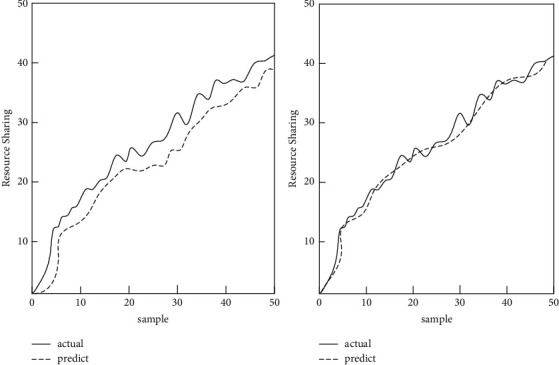
Fitting diagram of Naive Bayes model and BP neural network prediction.

In this experiment, under the setting of the same neural network structure, the fitting effect of the two models on historical data and the prediction results for a period of time in the future are, respectively, shown. It also compares the model's performance on datasets with significantly different lengths and features, as shown in Figure 10.

As shown in Figure 10, the BPNN has a very good fitting effect on historical data and can basically reproduce the real data. The fitting effect of the Naive Bayesian model on historical data is slightly worse than that of the BP neural network. It cannot accurately recover the short-term numerical fluctuations in historical data, but it can still grasp the trend of time series movement. The prediction results of the Naive Bayes model and the BP neural network on the data are shown in Table 4.

As shown in Table 4, the prediction error of the Naive Bayesian model is between 0.69 and 0.75, and the error of the BP neural network is between 0.21 and 0.43. The Naive Bayesian model has a bigger gap than the BPNN in the prediction results of time series data. With the increase of the prediction step, the Naive Bayes model will be limited to the periodicity of local fluctuations, and there will be periodic repeated numerical fluctuations. The BPNN model can basically predict the change of the shock trend in time.

## 5. Conclusions

The contradiction between the shortcomings and unbalanced development of the educational needs of colleges and universities has made all sectors of society focus more and more on this. This also makes the sharing of higher education resources an important requirement to promote the rapid development of China's higher education. Teachers rely on computers for education as a result of the advancement of modern computer technology, and the distribution of many software and assignments is dependent on networks and computers. This can improve resource sharing efficiency, expand educational resource content, and maximize the use of educational content. In recent years, the English major has grown in popularity, and major colleges and universities have increasingly focused on teaching English majors. This paper focuses on how to use teaching resource sharing responsibly and deliver effective instruction. The ubiquitous learning resource sharing platform is described in detail in this paper, and it is discovered that the platform has numerous advantages. It not only enables teachers to share resources at any time and from any location, but it is also very efficient. This paper briefly discusses the challenges of sharing teaching resources for English majors before highlighting the neural network's function. The neural network is used primarily for classification and prediction in this paper. The classification and prediction effects of the BP neural network are higher than those of the Bayesian classification algorithm, according to a series of experiments. There are still some errors in the data due to the experiment's technical and environmental factors. The author will continue to learn from his mistakes and strive to improve his work in the future.

## Figures and Tables

**Table 1:** Number and percentage of English learners from 2015 to 2018.

years	Number of people (thousands)	Percentage (%)	Effective percentage (%)
2015	56,213	37	37
2016	63,890	49	49
2017	67,583	52	52
2018	94,561	85	85

**Table 2:** Experimental data.

Data name	Data length	Number of training sets	Number of test sets	Number of categories
Beef	470	30	30	5
Gun-Point	150	20	150	2
Trace	270	20	100	4
ItalyPowerDemand	20	10	1000	2
Lightning-7	319	30	70	7
OSU Leaf	420	25	240	6
Wafer	150	10	6100	2
WordSynonyms	265	120	620	125
Yoga	420	10	2500	2

**Table 3:** Classification accuracy of different methods on different datasets.

Data name	Naive Bayes	Support vector machine classification algorithm	BP classification algorithm
Beef	0.89	0.750	0.932
Gun-Point	0.654	0.473	0.790
Trace	0.703	0.648	0.743
ItalyPowerDemand	0.926	0.732	0.964
Lightning-7	0.583	0.549	0.674
OSU Leaf	0.269	0.216	0.315
Wafer	0.894	0.809	0.932
WordSynonyms	0.237	0.251	0.367
Yoga	0.518	0.437	0.522

**Table 4:** Prediction results of the two algorithms.

Error	Naive Bayes model	BP neural network
MAE	0.75	0.43
MAPE	0.70	0.36
RMSE	0.69	0.21

## Data Availability

The data used to support the findings of this study are available from the author upon request.
